# Heterosis in COMT Val158Met Polymorphism Contributes to Sex-Differences in Children’s Math Anxiety

**DOI:** 10.3389/fpsyg.2019.01013

**Published:** 2019-05-15

**Authors:** Annelise Júlio-Costa, Aline Aparecida Silva Martins, Guilherme Wood, Máira Pedroso de Almeida, Marlene de Miranda, Vitor Geraldi Haase, Maria Raquel Santos Carvalho

**Affiliations:** ^1^Departamento de Psicologia, FAFICH, Universidade Federal de Minas Gerais, Belo Horizonte, Brazil; ^2^Programa de Pós-graduação em Neurociências, Instituto de Ciências Biológicas, Universidade Federal de Minas Gerais, Belo Horizonte, Brazil; ^3^Departamento de Biologia Geral, Instituto de Ciências Biológicas, Universidade Federal de Minas Gerais, Belo Horizonte, Brazil; ^4^Programa de Pós-Graduação em Genética, Departamento de Biologia Geral, Instituto de Ciências Biológicas, Universidade Federal de Minas Gerais, Belo Horizonte, Brazil; ^5^Instituto Nacional de Ciência e Tecnologia sobre Comportamento, Cognição e Ensino (INCT-ECCE), São Carlos, Brazil; ^6^Department of Neuropsychology, Institute of Psychology, University of Graz, Graz, Austria; ^7^Programa de Pós-Graduação em Psicologia: Cognição e Comportamento, Departamento de Psicologia, FAFICH, Universidade Federal de Minas Gerais, Belo Horizonte, Brazil; ^8^Programa de Pós-Graduação em Saúde da Criança e Adolescente, Faculdade de Medicina, Universidade Federal de Minas Gerais, Belo Horizonte, Brazil

**Keywords:** *COMT*, catechol-*O*-methyltransferase, heterosis, math anxiety, sex differences, dyscalculia

## Abstract

Math anxiety (MA) is a phobic reaction to math activities, potentially impairing math achievement. Higher frequency of MA in females is explainable by the interaction between genetic and environmental factors. The molecular-genetic basis of MA has not been investigated. The *COMT* Val158Met polymorphism, which affects dopamine levels in the prefrontal cortex, has been associated with anxiety manifestations. The valine allele is associated with lower, and the methionine allele with higher, dopamine availability. In the present study, the effects of sex and *COMT* Val158Met genotypes on MA were investigated: 389 school children aged 7–12 years were assessed for intelligence, numerical estimation, arithmetic achievement and MA and genotyped for *COMT* Val158Met polymorphism. The Math Anxiety Questionnaire (MAQ) was used to assess the cognitive and affective components of MA. All genotype groups of boys and girls were comparable regarding genotype frequency, age, school grade, numerical estimation, and arithmetic abilities. We compared the results of all possible genetic models: codominance (Val/Val vs. Val/Met vs. Met/Met), heterosis (Val/Met vs. Val/Val *plus* Met/Met), valine dominance (Val/Val *plus* Val/Met vs. Met/Met), and methionine dominance (Met/Met *plus* Val/Met vs. Val/Val). Models were compared using AIC and AIC weights. No significant differences between girls and boys and no effects of the *COMT* Val158Met polymorphism on numerical estimation and arithmetic achievement were observed. Sex by genotype effects were significant for intelligence and MA. Intelligence scores were higher in Met/Met girls than in girls with at least one valine allele (valine dominance model). The best fitting model for MA was heterosis. In Anxiety Toward Mathematics, heterozygous individuals presented MA levels close to the grand average regardless of sex. Homozygous boys were significantly less and homozygous girls significantly more math anxious. Heterosis has been seldom explored, but in recent years has emerged as the best genetic model for some phenotypes associated with the *COMT* Val158Met polymorphism. This is the first study to investigate the genetic-molecular basis of MA.

## Introduction

Math anxiety (MA) is a learned phobic reaction toward math activities that may importantly impair math learning (Dowker et al., 2016). MA is complex and manifests itself at different levels: cognitive (negative attitudes, worrisome rumination, feelings of helplessness, low self-esteem and self-efficacy, etc.); affective (dysphoria); behavioral (avoidance, hurry to finish math tasks, etc.); and physiological (sweating, trembling, high pulse rate, etc.) (Ashcraft et al., 2007). Although MA is a multidimensional construct, it is usually measured through self-report scales focusing on two dimensions: cognitive (performance perceptions and beliefs) and affective (emotional reactions and feelings) (Wood et al., 2012, see review in Haase et al., 2019).

In this study, we investigate the relevance of the *COMT* Val158Met polymorphism for sex differences in MA. In the Introduction, we will present the following topics: (a) sex differences in MA; (b) behavioral genetics of MA; (c) genetic models; (d) *COMT* Val158Met polymorphism and cognition; (e) *COMT* Val158Met polymorphism and anxiety; (f) outline of the present study.

Sex differences in math anxiety have already been described. MA levels are significantly higher in females than in males (Hembree, 1990; Dowker et al., 2016) and in certain professional categories, such as nurses and elementary school teachers (Hembree, 1990; Beilock et al., 2010; McMullan et al., 2012). Sex differences are observed from early school age on and tend to increase over time (Dowker et al., 2012). Possible societal consequences include less participation of females in math-intensive fields (Ceci et al., 2014).

Issues involving sex, math achievement and MA are complex. Low math achievement does not seem to be the cause of higher MA levels in females. Average math performance in males and females is highly similar. In recent years, a tendency of girls to obtain better grades in math than boys has been observed (Dowker et al., 2012). However, more males than females are found at the highest levels of math performance (Wai et al., 2010; Stoet and Geary, 2013).

Some possible experiential factors associated with higher rates of MA in females would be proneness and willingness to admit anxiety symptoms (Chapman et al., 2007; McLean et al., 2011), a sex stereotype threat (Spencer et al., 1999), and social transmission of MA by female teachers (Beilock et al., 2010) and parents (Eccles et al., 1990, see review in Gunderson et al., 2012). However, higher MA levels in girls and undervaluation of girls’ math abilities by parents seem to be independent of socioeconomic development and sex equity in cross-national comparisons (Stoet and Geary, 2015, 2016; Ireson, 2016). This may indicate the effects of female exposure to a more competitive environment or inherent affective/motivational differences between the sexes.

Much attention has been given to the gender stereotype threat as an important socio-cognitive mechanism underlying MA (Dowker et al., 2016). When women are reminded of the “males are better at mathematics than females” stereotype, their performance drops (Spencer et al., 1999). Neuroimaging studies indicate that the gender stereotype threat in math situations activates ventral cerebral areas associated with negative emotional processing and inhibits dorsal areas relevant to controlled and math processing (Krendl et al., 2008). However, Stoet and Geary (2012) observed that most studies only uncovered stereotype effects when prior math performance was statistically controlled. Therefore, as math performance is the outcome of interest, statistical control for prior math performance differences may confound between predictor and outcome. Stoet and Geary (2012) observed that only 55% of the studies replicated the original Spencer et al. (1999) finding, half of which adjusted for prior math achievement. Only 30% of studies without such adjustment reported significant effects of the stereotype threat.

In addition, neurocognitive differences could underlie MA sex susceptibility. This is supported by a study showing that lower MA levels in boys were mediated by better visuospatial processing abilities (Maloney et al., 2012). These subtle, but potentially relevant, cognitive differences could originate from fetal testosterone levels (Stoet and Geary, 2016). Supporting this hypothesis, a low negative correlation has been observed between 2D:4D digit-ratio, a marker of higher fetal testosterone levels, and related constructs such as math achievement and computer anxiety (de Bruin et al., 2006; Fink et al., 2006; Bull et al., 2010; Brosnan et al., 2011).

There are many hypotheses, and the origins of the higher female MA levels have been subject to considerable debate (Stoet and Geary, 2012). Overall, it is safe to conclude that both genetic and environmental factors contribute to the phenomenon. A diathesis-stress model could be advanced to explain sex differences in MA. According to this model, higher MA levels in females could be the result of interactions between specific neurocognitive vulnerabilities (such as fetal testosterone levels and yet to be discovered genetic influences) and environmental stress sources (such as low adult expectations and sex stereotype threat). Testing of this model requires a deeper understanding of the neurobiological, and especially the genetic, bases of MA. Understanding the neurogenetic underpinnings of MA susceptibility is essential for planning effective interventions.

Behavioral genetics of math anxiety have already been investigated. Two behavioral genetic studies investigated MA in twins (Wang et al., 2014; Malanchini et al., 2017). Heritability estimates were moderate (around 40%). Genetic correlations were observed with other forms of anxiety such as general anxiety and spatial anxiety. Both shared and non-shared environmental influences were uncovered. Wang et al. (2014) results suggest that MA emerges from the interaction between genetic influences on math performance and general anxiety. General anxiety, in turn, emerges from the interaction between genetic and non-shared environmental influences. Malanchini et al. (2017) obtained similar results, indicating a role for genetic and non-shared environmental factors, and for both shared and specific genetic influences on spatial anxiety and MA. No genetic or environmental sex-specific effects were investigated in these two studies.

To the best of our knowledge, no previous research has addressed the molecular-genetic underpinnings of MA. Other forms of anxiety have been associated with a host of genetic polymorphisms in several neurochemical systems (Stein et al., 2006). In this article, we focus on the dopaminergic system, as it has been implicated in various forms of performance anxiety (Mathew and Ho, 2006).

Another topic to consider is the genetic models to investigate. The impact of a specific genetic variation on a phenotype depends on the function of the protein or RNA considered. Most of the proteins are expressed from both alleles. As a consequence, the impact of genetic variants leading to aminoacid substitutions that change protein function depend on the genotype, meaning the pair of alleles present on an individual. For any locus having two alleles, say, allele 1 and allele 2, the effect, the effects depend on the genotype present, 11, 12, or 22. However, it also depends on the relationship between these alleles. Consider, for example an enzyme, being 1 the wild type allele and 2 a less functional allele.

**FIGURE 1:**
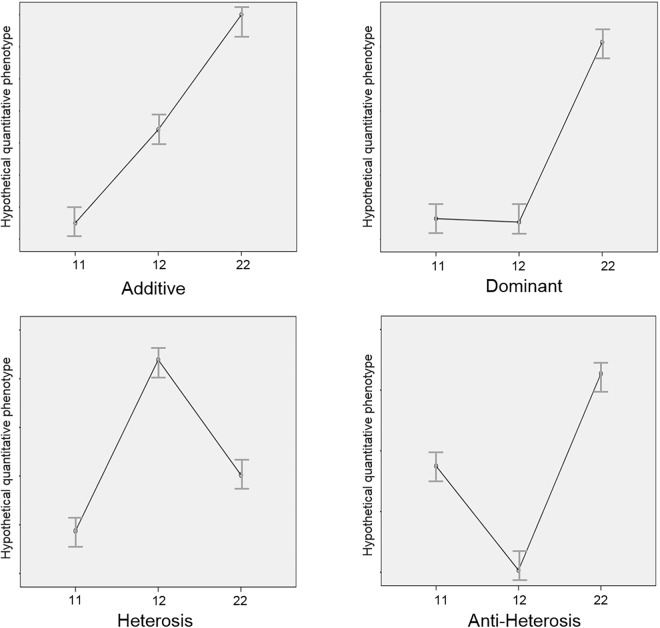
Graphic representation of the genetic models. *y*-axis: any hypothetical quantitative phenotype. *x*-axis: 1 and 2 are different alleles at the same locus and 11, 12, and 22 are the possible genotypes. The genetic models represent the different possible interactions between alleles in a specific genotype. Starting on the top, left, and going clockwise, figures represent examples of additive (codominance), dominance, anti-heterosis and heterosis models.

In an additive or codominance model, the genotype 11 would provide more enzyme activity, the genotype 12, less and the genotype 22 still less activity. In a 1 dominant model, 11 and 12 genotypes would produce similar enzyme function and 22 genotype would provide less (or more) enzyme activity. In the 2 dominant model, the effect would be the contrary. A third situation is seen when the both homozygous (11 and 22) genotypes produce similar enzyme activity and the heterozygous (12) genotype produces a different level of activity. When the heterozygous genotype is advantageous, the term heterosis is used. When the heterozygous genotype is disadvantageous, the term anti-heterosis is used. The term overdominance is also used, meaning heterosis. In Figure 1, we offer a graphic representation of these phenomena.

*COMT* Val158Met polymorphism has already been associated with cognition. Genetic polymorphisms in the catechol-*O*-methyltransferase (*COMT*) gene are a possible source of sex variability in cognitive and emotional processes, including math achievement and MA. The *COMT* Val158Met polymorphism (rs4680) has been particularly investigated. As a consequence of a nucleotide substitution in codon 158, a valine (Val) in position 158 of the protein is replaced by a methionine (Met). Three genotypes are thus defined: Val/Val, Val/Met and Met/Met, with consequences for the enzyme’s rate of catabolism. The presence of valine as compared to methionine is associated with higher COMT activity and lower dopamine availability at the synaptic cleft (Chen et al., 2004). This *COMT* polymorphism has been associated with several cognitive and emotional functions regulated by the prefrontal and parietal cortices, such as working memory (Goldberg et al., 2003; Mier et al., 2010; Júlio-Costa et al., 2014), numerical cognition (Tan et al., 2007; Júlio-Costa et al., 2013), impulsivity (Stein et al., 2006), anxiety (Mier et al., 2010; Gottschalk and Domschke, 2017), and psychiatric conditions such as schizophrenia (González-Castro et al., 2016), ADHD (Kebir and Joober, 2011; Bonvicini et al., 2016), autism (Nikolac Perkovic et al., 2014), etc.

Early results suggested that the valine allele would be associated with lower working memory performance and impulsivity (Stein et al., 2006; Mier et al., 2010; see also Dickinson and Elvevag, 2009). The methionine allele was, otherwise, implicated in higher working memory performance and anxiety. The connection between *COMT* Val158Met and numerical and arithmetic performance was explored in a study performed with typically developing children aged 7–12. The group with at least one methionine allele displayed more accurate non-symbolic number estimation (indexed by the coefficient of variation, cv), non-symbolic magnitude comparisons (indexed by the internal Weber fraction, *w*) and number transcoding (Júlio-Costa et al., 2013). Next, we discuss the association between the Val158Met COMT polymorphism and anxiety manifestations more specifically.

*COMT* Val158Met polymorphism and anxiety have also been associated. The association of the *COMT* Val158Met polymorphism with cognitive and emotional functions is subject to influences by culture, age and sex in adult samples (see reviews in Lee and Prescott, 2014; Barzman et al., 2015). The *COMT* Val158Met polymorphism has been implicated in anxiety manifestations in males and females (Hosák, 2007; Harrison and Tunbridge, 2008). Early reviews pointed out that both the valine and methionine alleles could be associated with anxiety-related phenotypes such as personality traits (e.g., neuroticism) and related disorders (e.g., generalized anxiety and panic disorder; Domschke et al., 2004; Harrison and Tunbridge, 2008). In these studies, interactions with sex were also extremely variable and complex, with a tendency for genotype-phenotype associations to be more salient in females.

More recent research also supports a nuanced picture of the association between *COMT* genotypes and anxiety manifestations. For example, Chen et al. (2011) found a COMT-by-sex interaction effect on affect-related personality traits in a large sample of the Chinese population. Males with at least one valine allele showed significantly higher scores on negative emotions than methionine homozygous males. Valine homozygous males presented lower scores on positive emotions, when compared with males possessing at least one methionine allele. A reverse tendency was observed in females, but the results were not significant. In another study, the Val158Met polymorphism was observed to interact with sex and neuroticism, but not with clinical symptoms of anxiety (Lehto et al., 2013). The interaction with neuroticism was investigated at three different ages (15, 18, and 25 years) in the same cohort. Valine homozygous females presented higher levels of neuroticism in the last assessment when compared to all other sex and genotype groups. Finally, females with at least one valine allele presented a tendency for higher levels of state and trait anxiety and lower reaction times than males, when viewing faces expressing fear or anger (Domschke et al., 2012). Statistically significant higher activation rates were observed using fMRI in the ventral visual stream, amygdala, and lateral prefrontal cortex in valine homozygous females, when compared with all other sex and genotype groups. These studies show that the associations between the effects of the *COMT* Val158Met polymorphism and anxiety-related manifestations are complex and moderated by sex.

Effects of the *COMT* val158met polymorphisms may interact with sex hormones. It has been shown that estrogen down-regulates COMT activity; i.e., this hormone reduces the rates of enzyme activity (Gogos et al., 1998; Xie et al., 1999; Jiang et al., 2003). Estrogen levels could then amplify the association between the valine allele and lower dopamine bioavailability in the synaptic cleft at the prefrontal cortex. A meta-analysis suggested complex interactions between the *COMT* Val158Met polymorphism and sex (Lee and Prescott, 2014). Valine homozygous males had higher neuroticism and/or harm avoidance than methionine homozygous males. No significant associations were found in women. Lee and Prescott (2014) criticize the current literature for not controlling the effects of menstrual phase and the use of hormonal birth control.

The complexity of the interactions between the *COMT* Val158Met polymorphism and sex is also reflected in studies with children and adolescents. In general, studies with children reveal that the *COMT* Val158Met polymorphism may act as a moderator between different kinds of anxiety manifestations in hetero-report measures and environmental stressors such as early emotional trauma and maternal anxiety. Some studies have implicated the methionine allele (Olsson et al., 2007; Baumann et al., 2013) and other studies have implicated the valine allele (Sheikh et al., 2013, 2017). A dose effect for the methionine allele was observed in Olsson et al. (2007) study. The number of methionine alleles was associated with higher risk for persistent episodic anxiety in females, but not in males.

However, other studies have reported negative results, failing to find either the involvement of the Val158Met polymorphism with anxiety or the interaction with sex (Evans et al., 2009). The current state of knowledge does not allow generalizations regarding the involvement of the *COMT* Val158Met polymorphism in anxiety, role of the alleles involved, interactions with other genes and hormones, or interactions with sex and age. This is illustrated in Supplementary Table S1, which presents the methods and results of the ten original articles reporting 11 studies, identified at PubMed in October 15th, 2018 using the key words “*COMT*” AND “anxiety” AND “child.” Fourteen out the 24 articles retrieved were excluded, because they were review articles, or did not investigate human subjects, did not focus on children, did not have comparison groups, or focused on psychotic and obsessive-compulsive disorder symptoms. One article reported results from two studies (Sheikh et al., 2013). Six of the 11 reported studies investigated the interaction between sex and *COMT* influences on anxiety.

The extant literature on effects of the sex by *COMT* Val158Met polymorphism on anxiety-related manifestations is scarce and extremely variable regarding age, anxiety measures, design, sampling, etc. Half of the six studies specifically examining this interaction obtained negative results. In only one of these studies with significant interactions, data were provided, from which a small effect could be estimated (*d* = 0.15) (Sheikh et al., 2017). From this literature, it is not possible to formulate more specific hypotheses on the *COMT* Val158Met polymorphism effects on anxiety-related manifestations that could eventually be applied to MA.

### Outline of the Present Study

As reviewed above, MA is a potential cause of under-representation of females in math-demanding careers. According to the diathesis-stress model of etiology, MA could result from the interaction of environmental and genetic factors. Some environmental factors, such as the low expectations of parents and teachers and the stereotype threat, have been extensively investigated. Neurobiological studies have focused on the possible role of fetal testosterone levels. No previous research has directly addressed the molecular-genetic underpinnings of MA and its sex differences.

In the current study, we investigate the impact of the *COMT* Val158Met polymorphism and sex on numerical estimation, math achievement and MA, searching for interactions between these variables in school-age children. To this end, we genotyped the *COMT* Val158Met polymorphism in a group of demographically recruited, school-age children, with intelligence scores above the PR10. We also assessed the children’s performance on tests of arithmetic achievement, numerical estimation and, in MAQ, an MA self-report questionnaire (Haase et al., 2012; Wood et al., 2012).

Studies investigating the association between the *COMT* Val158Met polymorphism and several anxiety forms have resulted in largely incongruent and inconclusive results. A source of incongruent results in association studies is the genetic models tested. Most association studies assume codominance (additive, multiplicative, etc.) or dominance models. Heterosis has been much less frequently tested when investigating the effects of a single locus. At a single locus level, heterosis has also been referred to as molecular heterosis (Comings and MacMurray, 2000, for a review). It refers to a situation in which the phenotype in heterozygous individuals differs from that of both homozygotes. Positive heterosis refers to higher performance in heterozygotes and negative heterosis refers to lower performance in heterozygotes. Heterosis (here meaning molecular heterosis) has been frequently described for some genes expressed in the brain, including the dopamine receptors and *COMT* (Comings and MacMurray, 2000; Gosso et al., 2008; Luijk et al., 2011). The term overdominance is used in the literature to imply that the hybrid vigor described in association with heterosis is effectively caused by heterozygote advantage, in opposition to epigenetic effects (Charlesworth and Willis, 2009, for a review).

In the present study, we investigate four different genetic models, representing the different possible interallelic interactions in a locus. First, in the codominance model, results of the three possible genotypes (Val/Val, Val/Met, and Met/Met) are compared. Second, in the heterosis model, the results of children having the heterozygous genotype (Val/Met) are compared to a group composed of the two homozygous genotypes (Val/Val *plus* Met/Met). Third, in the valine dominance model, results from children having at least one valine allele (meaning genotypes Val/Val *plus* Val/Met) are compared with the results of children having the Met/Met genotype. Fourth, in the methionine dominance model, the results of children with at least one methionine allele (Met/Met *plus* Val/Met genotypes) are compared to the results of children having the Val/Val genotype. To the best of our knowledge, this is the first study to investigate the molecular-genetic underpinnings of MA.

## Materials and Methods

### Participants

Participants were recruited from students in the 1st to 6th grades, enrolled in public and private schools in Belo Horizonte city, Brazil. Sampling was by convenience, respecting the proportion of 80% of children attending public schools, as observed in the city population. The sample covers the intermediate socio-economic strata of the Brazilian population (PR25 to PR75) (Associação Brasileira de Empresas de Pesquisa [ABEP], 2018). The sample comprised 389 children with ages ranging from 7 to 12 years (mean age = 115.66 [*sd* = 12.97] months, 55.32% female) and normal intelligence (PR > 10). Children participated only after informed consent was obtained in written form from parents and orally from themselves.

### Instruments

#### Raven’s Colored Progressive Matrices

General intelligence was assessed using the Raven’s Colored Progressive Matrices – CPM (Angelini et al., 1999). *z*-scores were calculated based on the manual’s norms.

#### Arithmetics Subtest of the Brazilian School Achievement Test (TDE)

This test is composed of three simple orally presented word problems (e.g., which is the largest, 28 or 42?) and 45 written arithmetic calculations of increasing complexity (e.g., very easy: 4-1; easy: 1230 + 150 + 1620; intermediate: 823 × 96; hard: 3/4 + 2/8). Specific norms for each school grade were used to characterize children’s performance (Stein, 1994; Oliveira-Ferreira et al., 2012). For the present study, the *z*-scores were calculated by grade.

#### Math Anxiety Questionnaire (MAQ)

The present study used a Brazilian Portuguese validated and standardized version (Haase et al., 2012; Wood et al., 2012). The MAQ items have the format of one out of four types of questions: “How good are you at…”; “How much do you like…”; “How happy or unhappy are you if you have problems with…” and “How worried are you if you have problems with…”. Each question is answered in regard to six different categories related to math, namely: mathematics in general; easy calculations; difficult calculations; written calculations; mental calculations; and, math homework. Children are encouraged with supportive figures to give their responses according to a 5-point Likert scale (coded 0 to 4). Responses for each kind of question are used to build the four MAQ subscales: MAQ A – Self-perceived Performance; MAQ B – Attitudes Toward Mathematics; MAQ C – Unhappiness About Mathematics; and, MAQ D – Anxiety Toward Mathematics, according to the authors of the original British version (Thomas and Dowker, 2000). The MAQ assumes that MA is a multidimensional construct. Scales MAQ A and MAQ B assess cognitive dimensions and scales MAQ C and MAQ D tap on the affective components of MA (Wood et al., 2012). The several subscales represent correlated but independent dimensions. The best structural description reduced the MAQ to two constructs, assessing the cognitive (MAQ AB) and the affective (MAQ CD) components of MA (Wood et al., 2012). The higher the score, the higher the MA level. In the present sample, Cronbach’s alpha coefficients were similar to those of the original report (Wood et al., 2012), varying from 0.76 (MAQ B) to 0.86 (MAQ Total). An age-standardized z-score was calculated for each MAQ scale.

#### Magnitude Estimation

In the non-symbolic magnitude estimation task, participants were asked to estimate, with a verbal response, the quantity of dots shown on the computer screen (Júlio-Costa et al., 2013; Pinheiro-Chagas et al., 2014). Black dots were presented in a white circle against a black background. The numerosities were 10, 16, 24, 32, 48, 56, or 64 dots. Each numerosity was presented 5 times, every time in a different configuration, such that the same numerosity never appeared in consecutive trials. The task comprised 35 testing trials. To avoid counting, the maximum stimulus presentation time was set to 1,000 ms. As soon as the child responded, the examiner, who was seated next to the child, pressed the spacebar on the keyboard and typed the child’s answer. Between individual trials, a fixation point appeared on the screen, which was a cross printed in white, with 3 cm for each line. To prevent the use of non-numerical cues, the sets of dots were generated using MATLAB in such a way that, in half of the trials, dot size remained constant and total dot area covaried positively with the numerosity; in the other half of the trials, total dot area was held constant and dot size covaried negatively with numerosity. Thus, neither total occupied area nor dot size could serve as cues for distinguishing between the different numerosities. To avoid memorization effects due to the repetition of a specific stimulus, on each trial, the stimuli were randomly chosen from a set of 10 precomputed images with the given numerosity. The data were trimmed for each subject, to exclude the responses 3 sd below or above the mean chosen value across all of the trials. As a measure of non-symbolic number representation acuity, we calculated the mean coefficient of variation (cv) of each child’s responses.

### Procedures

Data collection took place at the participants’ schools. At first, the intelligence test (Raven’s CPM) and the arithmetic subtest of the Brazilian School Achievement Test (TDE – Math) were applied in groups of eight children. This screening lasted approximately 40 min. Subsequently, parents were called to a meeting to collect the biological material (peripheral venous blood or saliva). Finally, children also answered the MAQ individually and performed the numerical magnitude estimation task in a quiet room (approximately 30 min). Data were collected from 395 children in the screening phase. Six children did not participate in the individual assessment because they performed below the PR10 on the Raven’s CPM.

### Genetic Analyses

DNA was extracted from peripheral blood or saliva using saline precipitation protocol (Miller et al., 1988). *COMT* Val158Met (rs4680) polymorphism was genotyped using two methods: (a) TaqMan SNP genotyping assay, genotyping was performed in ABI 7900 and analyzed using TaqMan Genotyper Software (Thermo Fisher Scientific, United States); (b) Tetra-primer amplification refractory mutation system-polymerase chain reaction (ARMS-PCR), as previously described by Ruiz-Sanz et al. (2007). In approximately 20% of the sample, genotyping was double-checked using PCR-RFLP with the restriction enzyme *Hsp*92II. This confirmed the results obtained through TaqMan SNP genotyping assay. These procedures are described in Júlio-Costa et al. (2013). Hardy–Weinberg equilibrium was tested using GenePop on the Web (Raymond and Rousset, 1995; Rousset, 2008). The predictive power sample of 80% by sex group was estimated using the Quanto software, considering an alpha = 0.05 (Gauderman, 2002, 2003).

### Statistical Analyses

Group differences in the distribution of sex, age, intelligence, school grade, arithmetic achievement, magnitude estimation, mathematics anxiety, as well as interactions with the *COMT* polymorphism, were examined. We explored the influence of intelligence on math achievement, numerical estimation and MA using correlation analysis, and the impact of sex using *t*-Student test. Since intelligence may confound the interpretation of possible interactions among sex, *COMT* polymorphism, school achievement and math anxiety, this variable (intelligence) was included as a covariate in further comparisons. The impact of the *COMT* polymorphism on school achievement and math anxiety was investigated by between-subjects analysis of covariance (ANCOVA).

To examine the interaction between *COMT* genotype, MA, and sex, we performed a four factorial ANCOVA using sex and *COMT* polymorphism as between-subjects factors, magnitude processing and arithmetic achievement as covariates; this procedure was repeated using each MAQ scale as the dependent variable. To test more specifically the *COMT* polymorphism effects, four different genetic models were assessed (i.e., codominance, heterosis, valine dominance, and methionine dominance). In the first model, the codominance in the *COMT* polymorphism was represented by a factor with three levels (homozygotes Val/Val, homozygotes Met/Met, and heterozygotes). In the second model, heterosis was represented by a factor with two levels (homozygotes vs. heterozygotes). In the third model, the dominance of valine was represented by a factor with two levels (Val carriers vs. non-carriers). In the fourth model, the dominance of methionine was represented by a factor with two levels (Met carriers vs. non-carriers). To establish which model accounts best for the data on MA, the Akaike Information Coefficient was calculated for each of the four models as well as the corresponding Akaike weights. A decision about the best fit was made based on the Akaike weights (Burnham and Anderson, 2003). The Akaike Information Criterion (AIC) is a simple index of the degree of disparity between a statistical model and the empirical data. The lower the value of the AIC, the better the model depicts features of the data. The AIC utilizes information on the log-likelihood of each model as well as the number of model parameters, and penalizes models with higher complexity. It is a useful tool for comparing different statistical models. When considering a set of alternative models with their respective AIC values, the Akaike weights can be calculated. These indicate, as a proportion value, how much better a model is in comparison to alternative models (see Wagenmakers and Farrell, 2004 for a primer). Statistic tests were considered significant when values of *p* < 0.05 were observed.

## Results

Allele frequencies observed were Met: *n* = 310 (40%) and Val: *n* = 468 (60%). Genotype frequencies for the *COMT* Val158Met polymorphism in the sample are consistent with the Hardy–Weinberg equilibrium (*p* = 0.49). Participants were assigned to one of three groups according to their genotypes: (1) homozygous children for the valine allele (Val/Val): *n* = 141 (36.2%), (2) heterozygous children (Val/Met): *n* = 186 (47.8%) and (3) homozygous children for the methionine allele (Met/Met): *n* = 62 (15.9%). Proportions of boys and girls, their age, intelligence, grade, numerical magnitude estimation, arithmetic achievement, and MA scores are comparable [χ^2^(1) = 0.14; *p* = 0.93; η^2^ = <0.001] across the three *COMT* genotypes (Table 1). For a predictive power of 80%, the required sample size is 147 boys and 195 girls. Our sample is composed of 174 boys and 215 girls, evidencing that the sample has enough power to detect differences in MAQ-D between the genotypic groups considering sex.

**Table 1:** Demographic data of children divided according to sex and genotype.

	Sex	Total *n*(%)	Val/Val *n*(%)	Val/Met *n*(%)	Met/Met *n*(%)
Sample distribution	Girls	215(55)	79 (37)	103 (48)	33 (15)
	Boys	174 (45)	62 (36)	83 (48)	29 (17)

		**Mean (*sd*)**	**Mean (*sd*)**	**Mean (*sd*)**	**Mean (*sd*)**

Grade	–	4.0 (1.0)	4.0 (0.9)	4.0 (1.0)	4.1 (1.1)
Age (months)	Girls	116 (12)	116 (13)	117 (12)	117 (13)
	Boys	115 (14)	116 (13)	114 (15)	114 (16)
Coefficient of variation in magnitude estimation (*z*-score)	Girls	0.19 (0.07)	0.18 (0.07)	0.19 (0.07)	0.20 (0.07)
	Boys	0.20 (0.07)	0.19 (0.06)	0.19 (0.07)	0.21 (0.06)
Intelligence (*z*-score)	Girls	0.71 (0.8)	0.61 (0.8)	0.60 (0.8)	0.93 (0.7)
	Boys	0.80 (0.7)	0.89 (0.7)	0.82 (0.7)	0.53 (0.9)
Arithmetic achievement (*z*-score)	Girls	0.29 (1.1)	0.23 (1.1)	0.19 (1.1)	0.45 (1.1)
	Boys	0.20 (1)	0.31 (1)	0.14 (1)	0.14 (1)
MAQ A – Self-perceived Performance (*z*-score)	Girls	0.06 (0.94)	0.11 (0.92)	0.06 (0.96)	-0.04 (0.94)
	Boys	-0.08 (1)	-0.24 (1.09)	0.04 (0.97)	-0.09 (0.79)
MAQ B – Attitudes Toward Mathematics (*z*-score)	Girls	0.00 (0.9)	0.05 (0.94)	-0.05 (0.91)	0.01 (0.81)
	Boys	0.01 (1.03)	-0.07 (1.01)	0.13 (1.03)	-0.13 (1.06)
MAQ C – Unhappiness About Mathematics (*z*-score)	Girls	.01 (0.99)	0.00 (1.11)	-0.06 (0.98)	0.27 (0.91)
	Boys	-0.02 (0.92)	0.04 (0.85)	-0.02 (0.9)	-0.11 (0.99)
MAQ D – Anxiety Toward Mathematics (*z*-score)	Girls	0.09 (0.99)	0.24 (1.05)	-0.04 (0.95)	0.17 (0.95)
	Boys	-0.11 (0.92)	-0.31 (0.91)	0.01 (0.83)	-0.05 (1.14)

Missing data were restricted to the variable coefficient of variation (cv) of the numerical estimation task. In total, 16% of the values were missing. A chi-square test revealed that the proportion of missing values when considering sex and genotype was comparable [χ^2^(1) = 2.26, *p* = 0.132]. Correlation coefficients of coefficient cv (numerical estimation), arithmetic achievement and intelligence with MA were calculated (Table 2). Intelligence was positively correlated with arithmetic achievement and numerical estimation, and negatively, with MAQ B – Attitudes Toward Mathematics (one of the cognitive components of MA as assessed by MAQ). Numerical estimation correlated negatively with intelligence and arithmetic achievement, and positively with MAQ A – Self-perceived Performance. In turn, arithmetic achievement also correlated negatively with MAQ A – Self-perceived Performance, MAQ B – Attitudes Toward Mathematics, and MAQ C – Unhappiness about Mathematics. All MA subscales correlated positively with each other.

**Table 2:** Correlation coefficients between cognitive variables and mathematics anxiety.

*N* = 327	Coefficient of variation	Intelligence (z-score)	Arithmetic achievement	Attitudes Toward Mathematics	Unhappiness About Mathematics	Anxiety Toward Mathematics
Intelligence (*z*-score)	-0.170^*^					
Arithmetic achievement	-0.187^**^	0.414^*^				
MAQ A – Attitudes Toward Mathematics	0.102	-0.124^*^	-0.343^*^			
MAQ B – Unhappiness About Mathematics	0.163^**^	0.006	-0.198^**^	0.567^*^		
MAQ C – Anxiety Toward Mathematics	0.077	0.051	-0.018	0.317^**^	0.363^*^	
MAQ D – Self-perceived Performance	0.142^**^	0.068	-0.145^**^	0.284^**^	0.235^**^	0.556^*^

*^∗^p < 0.05, ^∗∗^p < 0.01.*

We calculated the impact of the *COMT* Val158Met polymorphism on the cv of numerical estimation, arithmetic achievement, and intelligence using ANOVA models with sex and the genetic models (i.e., codominance, heterosis, valine dominance, and methionine dominance) as between-subject factors, and compared the model fit using the AIC and AIC weights. Sex and *COMT* Val158Met polymorphism had no effect on numerical estimation, as no main- or interaction-effect reached significance (all *p* > 0.2). Sex and *COMT* Val158Met polymorphism also had no effect on arithmetic achievement (all *p* > 0.3). Importantly, an effect of *COMT* Val158Met polymorphism on intelligence was observed. The genetic model of valine dominance reached the smallest AIC (df = 5, AIC = 896) and the highest AIC weight (83%). All other models presented AIC values > 900 and Akaike weights < 14%. In the valine dominance model, a significant interaction for sex by genotype was observed [*F*(1,385) = 9.40, *p* = 0.002, η^2^ = 0.023]. None of the main-effects reached significance. Tukey *post hoc* tests revealed higher intelligence scores in Met/Met girls than in girls with at least one valine allele (*p* = 0.02).

**FIGURE 2:**
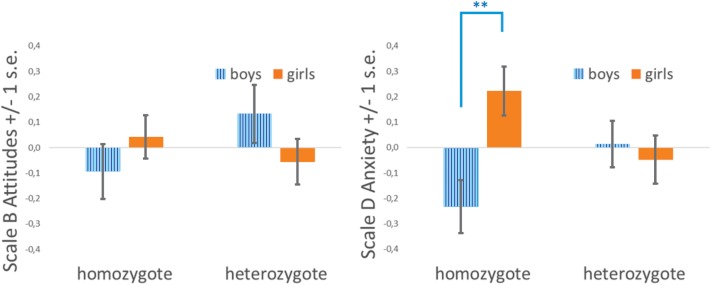
Levels of math anxiety in boys and girls as a function of *COMT* Val158Met genotype (heterosis model). (Left) MAQ B – Attitude Toward Mathematics. MAQ D – Anxiety Toward Mathematics (^∗∗^ Tukey HSD test adjusted for multiple comparisons, *p* < 0.005).

Genetic models (i.e., codominance, heterosis, valine dominance, and methionine dominance) were compared in order to determine the contribution of valine and methionine alleles to the sex-specific phenotypes of MA. The genetic models were evaluated using four different ANCOVA models in which numerical estimation and arithmetic achievement were entered as covariates. Intelligence was not included as a covariate, for statistical reasons (Miller and Chapman, 2001), since it is also associated with the *COMT* Val158Met polymorphism. Model fit was compared using the AIC and AIC weights. No genetic effects were observed on MAQ A – Self-perceived Performance or MAQ C – Unhappiness about Mathematics, as no main effect of sex or genetic model effect reached significance. In contrast, the interaction of sex by genotype was significant for MAQ B – Attitudes Toward Mathematics and MAQ D – Anxiety Toward Mathematics. Attitudes Toward Mathematics was better explained by a heterosis model (Table 3). Although the interaction of sex by genotype in the heterosis model reached significance, Tukey *post hoc* comparisons did not reveal any significant difference in pairwise comparisons (all *p* > 0.4, Figure 2).

**Table 3:** Results and comparison of the genetic models for the COMT polymorphism on scale MAQ B – Attitudes Toward Mathematics.

Scale: MAQ B – Attitudes Toward Mathematics	Df	SS	MS	*F*-value	*P*	$\eta_{p}^{2}$	AIC/wAIC
Sex	1	0.03	0.03	0.03	0.86	0.0	910(65%)
Heterosis	1	0.54	0.54	0.60	0.45	0.0	
*z*-mean cv of magnitude estimation	1	9.30	9.30	10.06	0.002	0.03	
*z*-arithmetic achievement	1	9.02	9.02	9.75	0.002	0.03	
Sex^∗^heterosis	1	5.20	5.20	5.62	0.02	0.02	
Residuals	321	292.35	0.93				

*The other models presented AIC in the interval (913.5–913.8) and AIC weights in the interval (10.8–13%).*

MAQ D – Anxiety Toward Mathematics was also better explained by the heterosis model (Table 4). Tukey *post hoc* comparisons revealed significant differences in pairwise comparisons between homozygous boys and girls (*p* < 0.005), but not between the heterozygous boys and girls. No other pairwise comparisons reached significance. Homozygous boys were significantly less anxious than homozygous girls, but heterozygous children were equally anxious regardless of their sex (Figure 2). MAQ D – Anxiety Toward Mathematics of heterozygous children were closer to the grand average.

**Table 4:** Results and comparison of the genetic models for the COMT polymorphism on scale MAQ D – Anxiety Toward Mathematics.

Scale: Maq D – Anxiety toward Mathematics	Df	SS	MS	*F*-value	*p*	$\eta_{p}^{2}$	AIC/wAIC
Sex	1	3.02	3.02	3.30	0.07	0.0	907 (72%)
Met dominance	1	0.01	0.01	0.01	0.91	0.003	
*z*-mean cv of magnitude estimation	1	6.37	6.37	6.96	0.01	0.02	
*z*-arithmetic achievement	1	5.00	5.00	5.46	0.02	0.02	
Sex^∗^Met dominance	1	11.20	11.20	12.24	0.0005	0.04	
Residuals	321	293.74	0.915				

*The other models presented AIC in the interval (910–918) and AIC weights in the interval (0–15%).*

## Discussion

In the present study, the effects of sex and *COMT* Val158Met genotypes on MA were examined in a large sample of boys and girls. No deviation from the Hardy–Weinberg Equilibrium expectancy was detected, implying that eventual differences between genotype groups do not reflect abnormalities in population genetic structure. The proportion of boys and girls in each genotype group was comparable. All genotype groups of boys and girls were comparable regarding their ages, school grades, number processing, and arithmetic abilities. Moreover, no significant differences were observed between girls and boys regarding numerical estimation or arithmetic achievement.

Intelligence was correlated positively and moderately with arithmetic achievement, and negatively and weakly with numerical estimation. Regarding MA, intelligence was negatively and weakly correlated only with the subscale MAQ B – Attitudes Toward Mathematics.

Correlations between MA and numerical/arithmetic tasks were observed. Numerical estimation correlated positively with MAQ A – Self-perceived Performance. Arithmetic achievement correlated negatively and weakly with all MA components except for MAQ D – Anxiety Toward Mathematics.

No associations between the *COMT* Val158Met polymorphism on numerical estimation and arithmetic achievement were observed. A sex by genotype interaction was observed for intelligence. Intelligence scores were higher in Met/Met girls than in girls with at least one valine allele (valine dominance model).

Our main result is related to the genetic models explaining MA. The best fitting model in both MAQ B - Attitudes Toward Mathematics and MAQ D – Anxiety Toward Mathematics was heterosis. In the case of MAQ B – Attitudes Toward Mathematics, no *post hoc* pairwise comparisons reached significance. In contrast, in the MAQ D – Anxiety Toward Mathematics scale, homozygous boys were significantly less anxious than girls, but heterozygous children were equally anxious regardless of their sex; heterozygous individuals reported MA levels close to the grand average.

In the next sections, we discuss the validity of our results, the effects of the *COMT* Val158Met polymorphism on general and numerical cognitive measures and on MA. We conclude by discussing the importance of heterosis as an explanatory model for the effects of the *COMT* Val158Met polymorphism on several cognitive-behavioral phenotypes, including MA.

### Number Processing, Arithmetic Achievement, and Intelligence

The cognitive and math-related performances of children observed in the present study were in line with data reported in the literature (Dowker et al., 2016). Intelligence was positively and moderately correlated with arithmetic achievement. Similar results have been consistently observed in other studies and intelligence is considered one of the best predictors of math achievement (Pind et al., 2003; Rohde and Thompson, 2007; Primi et al., 2010; Costa et al., 2011).

Intelligence was also negatively and weakly correlated with numerical estimation. Correlations on the same order of magnitude were observed in a large representative sample by Tosto et al. (2017). Theoretically, no correlation, or only weak correlations, might be expected, as the approximate number system (ANS) underlying numerical estimation, is usually understood to be a modular system relatively independent from general intelligence (Dehaene, 1992; Mandelbaum, 2013). However, additional evidence casts doubt on this assumption. Correlations between several tasks tapping the ANS, such as verbal estimation and symbolic and non-symbolic magnitude comparisons, are weak (Pinheiro-Chagas et al., 2014; Tosto et al., 2014). General cognitive factors (e.g., inhibitory executive functions) play a role in some ANS-related tasks, such as non-symbolic number comparison (Gilmore et al., 2013; Szûcs et al., 2013). Finally, in a large longitudinal study, age-varying patterns of predictive association were observed between prior general cognitive abilities and numerical estimation at age 16 (Tosto et al., 2017). Summing up, our results agree with the hypothesis that general cognitive requirements are important in the performance of numerical estimation tasks.

Arithmetic achievement was negatively and weakly correlated with numerical estimation, as observed by Tosto et al. (2017). Significant but small differences in verbal numerical estimation between children with and without math learning difficulties have been reported (Mejias et al., 2012; Pinheiro-Chagas et al., 2014). These results support the general view that basic numerical abilities, such as non-symbolic magnitude estimation, may be a precursor of the more advanced arithmetic abilities acquired during formal education (Piazza et al., 2010; Ferreira et al., 2012; Siegler and Braithwaite, 2017). However, the existence and strength of these associations may vary with age, tasks and domains of math assessed (Tosto et al., 2017).

Boys and girls were comparable regarding their ages, school grades, numerical estimation and arithmetic abilities independently of their genotype groups. No significant differences were observed between girls and boys in numerical estimation or arithmetic achievement. Therefore, differences in numerical estimation or arithmetic performance cannot account for the impact of the *COMT* Val158Met polymorphism on MA.

Interestingly, higher intelligence observed in Met/Met girls yields no higher arithmetic achievement in this group which, at the first glance, seems to be counterintuitive. As discussed below, Met/Met girls have higher MA levels, which could reduce the impact of their general intellectual advantage on arithmetic achievement.

### Math Anxiety

All MA subscales were positively correlated. This is in line with the literature pointing out that the four subscales of the MAQ represent different facets of the MA construct (Krinzinger et al., 2007; Wood et al., 2012), which are relatively independent from intelligence (Hembree, 1990). Accordingly, with the exception of MAQ B – Attitudes Toward Mathematics, no MAQ subscale correlated with intelligence. MAQ B – Attitudes Toward Mathematics exhibited a weak negative correlation with intelligence, which corroborates previous findings (Minato and Yanase, 1984; Moenikia and Zahed-Babelan, 2010) since, in the MAQ B scale, higher scores code for more negative attitudes toward mathematics.

Arithmetic achievement was negatively and weakly correlated with all MAQ scales, except for MAQ D – Anxiety Toward Mathematics. These results are also in line with previous studies (Moenikia and Zahed-Babelan, 2010). Since correlations between arithmetic achievement and MA are more pronounced in the subscale measuring the affective component of MA (Krinzinger et al., 2007; Haase et al., 2012; Wood et al., 2012), the effects of sex by *COMT* genotype interactions on MA seem to be emotionally mediated.

Our study was not designed to answer the question of the specificity of results regarding MA, as we did not use measures of more generalized anxiety or reading/spelling performance. MA is a complex construct, including both cognitive and affective dimensions (Dowker et al., 2016; Haase et al., 2019). Behavioral genetic models have shown that MA shares considerable sources of genetic and environmental influences with other anxiety-related constructs (Wang et al., 2014; Malanchini et al., 2017). However, correlations between MA and other forms of anxiety are usually weak (*r* = 0.3) (Hembree, 1990), suggesting that MA and other forms of anxiety represent partially independent dimensions. In a previous study using MAQ in school-aged children, we observed that correlations with generalized anxiety (assessed by CBCL) were weak, and that MAQ levels were associated with math performance but not with word spelling performance (Haase et al., 2012). The reverse pattern was observed for generalized anxiety. Generalized anxiety was associated with spelling but not with math performance. Considering the behavioral genetic results, it is safe to conclude that the construct MA refers to the content of phobic reactions in predisposed individuals.

Finally, differences in the covariance structure of MA in children with different genotypes are possible but remain elusive in the present study. This is because our sample size is not large enough for a useful estimation of correlations coefficients for different groups separately, particularly when considering only the boys or only the girls with the Met/Met genotype.

### *COMT* Val158Met Polymorphism and Cognition

No main or interaction effects of the factors sex and *COMT* polymorphism on basic magnitude estimation or arithmetic achievement were observed in the present study.

A link between dopaminergic activity and magnitude processing was established in experimental research in rodents. In rodents, pharmacological inhibition or facilitation of dopaminergic activity modulates temporal and numerical magnitude estimation (Cordes et al., 2007; Coull et al., 2011). Dopaminergic activity is related to the speed of the counting mechanism underlying magnitude estimation according to the accumulator model (Leslie et al., 2007). In humans, one study from our research group investigated the impact of the *COMT* Val158Met polymorphism on basic number processing tasks (Júlio-Costa et al., 2013). In that study, children with at least one methionine allele presented better performance in the numerical estimation and other numerical and arithmetic tests. The discrepancy between that study and the present one is only apparent. A large proportion of the sample assessed by Júlio-Costa et al. (2013) was also included in the present study. Therefore, disappearance of the effect with increase of sample size is indicative of a false positive result, probably caused by the smaller sample investigated in that study. The sample size of 327 children, for whom data were available on cv in the current report, offers a higher degree of protection against false positive findings and may be given more weight than the partial evidence published previously. Accordingly, evidence for a detectable impact of the *COMT* Val158Met polymorphism on basic magnitude estimation remains elusive, since the positive evidence obtained in rodents using pharmacological manipulations are much stronger than the functional differences occurring naturally between the valine and methionine containing enzyme.

Beyond the scope of basic magnitude estimation, sex and *COMT* Val158Met polymorphism also seem to have no impact on arithmetic achievement. In a small study using fMRI, Tan et al. (2007) explored the role of the *COMT* Val158Met genotypes in numerical/arithmetic processing. Adult carriers of the valine allele had higher levels of dorsolateral prefrontal cortex activation than individuals with other genotypes. This activation correlated with arithmetic operations that require working memory, but not with the operations requiring long-term memory retrieval. The increased brain activation during resolution of arithmetic problems in individuals with the valine allele may be interpreted as a compensatory mechanism (Tan et al., 2007). Consistent with the present study, however, no effects of genotype were observed at the behavioral level.

The connection between the *COMT* Val158Met polymorphism and numerical/arithmetic performance could also be investigated in 22q11.2 microdeletion syndrome (22q11.2DS). Individuals with 22q11.2DS present several phenotypic traits such as risk of schizophrenia, intellectual disability and math learning difficulties in the presence of hemizygosis at the *COMT* Val158Met locus (Karayiorgou et al., 2010). Some research supports a role for the valine allele in intellectual disability and schizophrenia (Shashi et al., 2006, 2010), but results have not always been replicated (Campbell et al., 2010; Franconi et al., 2016). However, to the best of our knowledge, the specific association between *COMT* Val158Met polymorphism and numerical/arithmetic abilities has not yet been investigated in 22q11.2DS.

A sex by genotype interaction was detected for intelligence. Met/Met girls exhibited higher intelligence scores compared to girls with at least one valine allele (valine dominance). The methionine allele is associated with higher intelligence in some studies (Enoch et al., 2009; Carmel et al., 2014), higher cognitive performance, and also higher anxiety levels (Stein et al., 2006; Dickinson and Elvevag, 2009). Specifically, Ramirez et al. (2013) showed that MA is higher on the extremes of the distribution of working memory capacity. Since Met/Met girls generally present higher cognitive ability, they would also be more affected by MA, as observed in the present study. One possible mechanism of how higher levels of MA may impair arithmetic achievement has been proposed by Ramirez et al. (2013). According to them, high performing individuals tend to rely on working memory-intensive solution strategies, which are likely disrupted when MA interferes with working memory. Therefore, Met/Met girls have high levels of intelligence but also high MA levels, which could reduce their general intellectual advantage on arithmetic achievement. These findings suggest a sex-specific connection between higher cognitive abilities, the Met/Met genotype, and susceptibility to interference of MA on math performance, which will be explored below in further detail. However, this connection between high intelligence and MA in girls is not the whole story, since Val/Val girls were not more intelligent than other groups of children in our study. Interestingly, evidence indicates higher levels of neuroticism in Val/Val women from adolescence to young adulthood (Lehto et al., 2013). Therefore, there is evidence of higher levels of anxiety in both homozygous genotypes of female participants. These pieces of evidence also will be discussed in further detail in relation to the heterosis model in the next section.

### *COMT* Val158Met Polymorphism and MA

Sex and *COMT* polymorphism had a marginal effect on subscale MAQ B – Attitudes Toward Mathematics, which represents a more cognitive aspect of MA. Here, it is important to consider the young age of the participants in our study. It is possible that their self-concept and attitudes toward mathematics were not yet as fully developed as later in puberty and adulthood. Stronger effects of sex on the cognitive aspect of MA are known to become more evident in older adolescents (Utsumi and Mendes, 2000; Mata et al., 2012). A specific interaction between sex and grade was obtained by Wigfield and Meece (1988). These authors assessed the cognitive and affective dimensions of MA in 564 children from 6th to 12th grades. Grade differences were observed only in the cognitive dimension, with older children scoring higher than younger ones. Sex differences were observed only in the affective dimension of MA, with girls scoring higher. No sex by grade interactions were observed (Wigfield and Meece, 1988).

In our study, more robust effects were observed in the subscale MAQ D – Anxiety Toward Mathematics, which represents a more affective aspect of MA. The interaction between sex and the *COMT* Val158Met genotype in MAQ D – Anxiety Toward Mathematics was significant under the heterosis model. Significant differences between boys and girls were observed in both homozygous groups, but not in heterozygous individuals. Homozygous girls presented higher levels of MA than boys, while heterozygous boys and girls did not differ regarding MAQ D – Anxiety Toward Mathematics. The existing literature on sex-related differences in anxiety levels associated with the Met/Met and Val/Val genotypes (reviewed in Supplementary Table S1) suggests different explanations for the higher levels of MA observed in Met/Met or in Val/Val girls. Our comparison of genetic models suggests that these apparently contradictory results may reflect the fact that the heterosis model has not been tested. The bulk of the literature on the *COMT* Val158Met polymorphism focuses on statistical models separating all three genotypes (codominance or additive models) or genotypes organized in two groups (dominance models). Therefore, cases in which heterosis is the correct genetic model for the data may have been easily overlooked; and, the number of explanations for the phenotypes connected with the different genotypes may be artificially inflated.

The genetic heterosis model of MAQ D – Anxiety Toward Mathematics suggests that homozygous girls are more susceptible than boys to the emotional arousal elicited by math tasks perceived as difficult. Whether the causal pathways of the genetic effects of Met/Met and Val/Val genotypes are the same or not is an open question. This can be answered only with more detailed studies. In the final two sections, we are going to discuss: (a) the mechanisms of estrogen effects that may contribute to increase the levels of MAQ D – Anxiety Toward Mathematics in homozygous girls; and (b) the role of heterosis in the *COMT* Val158Met polymorphism.

### Mechanisms of Estrogen Effects on *COMT*

Sex differences in many behavioral traits in humans have been described and attributed to the influence of sex hormones through their influences on neurotransmitter systems, such as dopamine (Sherwin, 2007; Riccardi et al., 2011; for an overview of dopamine system, see Wahlstrom et al., 2010).

The increase in the estrogen levels during puberty down-regulates *COMT* transcription and leads to sex differences in COMT enzyme activity (Xie et al., 1999; Tunbridge, 2010). As a consequence, females show higher dopamine levels in the synaptic cleft in brain regions where COMT is the main metabolizer of dopamine. Here, it is important to remember that dopamine can be depleted from the synaptic cleft by *DAT1* or by *COMT*. Consequences of *COMT* malfunctioning are more prominent in those regions where *DAT1* is physiologically poorly expressed, such as the prefrontal cortex. Therefore, functional *COMT* polymorphisms have a strong impact in cognitive tasks associated with attention and executive functions (Riccardi et al., 2006, 2011).

There is another interaction mechanism between *COMT* and estrogen. *COMT* metabolizes catechol estrogens (i.e., 2-OHE2, 2-OHE1, 4-OHE2, and 4-OHE1) to methyl-estrogen, which has been associated with cancer development and progression (Dawling et al., 2001; Ashton et al., 2006). These pathways have not yet been investigated in relation to cognitive functions.

The relationship between estrogen and *COMT* is even more complex. Men have 17% higher COMT activity in the prefrontal cortex than women, independently of any polymorphisms (Chen et al., 2004). Higher COMT activity in men has been described in most tissues (reviewed by Harrison and Tunbridge, 2008). Evidence for sexual dimorphism in *COMT* associated phenotypes is abundant, but frequently conflicting, in the literature. This suggests that a “dopaminergic tonus” or “optimal dopamine level” may differ according to sex, age, brain region or system, physiological or pathological state as well as pharmacological responses (Jacobs and D’Esposito, 2011).

These effects are clear in the evaluation of the impact of the *COMT* Val158Met polymorphism. For example, the association of the Met allele with obsessive-compulsive disorder in men but not in women (Karayiorgou et al., 1997) is a well replicated finding (Pooley et al., 2007). The Met allele has been associated with increased levels of anxiety and cautious personality (Enoch et al., 2003; Olsson et al., 2005; Montag et al., 2012) (see Supplementary Table S1), and anxiety disorder (Domschke et al., 2004; Woo et al., 2004; Rothe et al., 2006).

However, it is important to consider that physiological sex differences occur in many systems, not only in sex hormones. As a consequence, effects attributed to differences in sex hormones may reflect differences in other, less investigated biological systems.

*COMT* genotype and sex hormone influence may interact epigenetically in complex ways. In this sense, it is also important to consider that our participants were prepubertal children. Investigations with post-pubertal participants should follow the recommendations of Lee and Prescott (2014) to consider menstrual cycle phase and use of oral anticonceptives.

### Evidence for Heterosis in the *COMT* Val158Met Polymorphism

As mentioned above, the interaction between the alleles may take four main forms: codominance, heterosis, Val dominance and Met dominance. The term codominance is used to refer to situations in which the three genotypes have different effects at the phenotype level. The most used codominance model is the additive, in which each allele substitution has a incremental effect (e.g., considering a locus with two alleles, 1 and 2, the effects of the genotypes would be 11 < 12 < 22). In the heterosis model, heterozygous individuals have a phenotype that differs from both homozygous groups, which have similar phenotypes. The phenotype in heterozygous individuals can be advantageous (positive heterosis) or disadvantageous (negative heterosis).

Several examples of heterosis in the *COMT* Val158Met polymorphism have been reported already. We will discuss only those studies with substantial sample sizes. Barnett et al. (2007) investigated the effects of the *COMT* Val158Met polymorphism on working memory, verbal and motor inhibition, attentional control, and IQ in a sample composed of 8,707 children, aged 8–10 years. These authors described heterozygous advantage in a measure of sustained attention in boys but not in girls.

Gosso et al. (2008) described an example of positive heterosis in working memory. These authors investigated a sample of over 600 participants, approximately half of them children. Positive heterosis was detected: better results in working memory tests were found in Val/Met individuals who presented also the *DRD2* A1 allele, demonstrating also a gene-gene interaction.

Luijk et al. (2011) investigated the association of several genetic polymorphisms and infant attachment security and disorganization in a sample composed of over 500 children from two different cohorts. *COMT* Val158Met heterozygotes were more disorganized in both samples (combined effect size *d* = 0.22, CI95 = 0.10–0.34, *p* < 0.001), which the authors considered an example of negative heterosis.

Costas et al. (2011) investigated the hypothesis of overdominance (a. k. a., heterosis) in two samples of persons having schizophrenia (*n* = 762) and controls (*n* = 1,042). In these samples, they detected a protective effect against schizophrenia of the *COMT* Val/Met heterozygous genotype (OR = 0.75, CI95 = 0.62–0.91, *p* = 0.003). In addition, they conducted a meta-analysis including 13,894 schizophrenic patients and 16,087 controls from 51 studies. A protective effect of the Val/Met genotype was also detected (pooled OR = 0.946, CI95 = 0.904–0.989, *p* = 0.015).

It is important to consider that heterosis is by far the less investigated hypothesis regarding *COMT* effects on behavior. The wild-type allele at the 158 position is a valine. The mutation Val158Met is an evolutionary novelty present in the human, but not in the gorilla, chimpanzee, bonobo, and orangutan (Piffer, 2013). Currently, the frequency of the Met allele is usually high (20–60%; Piffer, 2013) in most of the populations reported so far. This is surprising, considering that the enzyme activity is importantly reduced by the Met allele. The high frequency of the Met allele suggests that some selection mechanism is in place. From the literature review presented here, two main possible mechanisms emerge. The Met allele may have reached high frequencies because Met/- genotypes are advantageous for some *COMT* related phenotypes. Alternatively, heterosis itself is advantageous because intermediate dopamine levels at the synaptic cleft would be more adaptive, under usual environmental conditions, than high or low levels (Arnsten, 1998). The same could happen in the case of MA. Our data suggest a positive heterosis model. Heterozygous individuals exhibit MA levels closer to the grand average, and are less susceptible to worries related to math performance. Males having both homozygous phenotypes present lower MA than all other groups. Females having both homozygous phenotypes present higher levels of MA than all other groups.

It is important to note some limitations in our study. First, the specificity of the MA construct could not be investigated, as we did not include measures of achievement in other domains (such as reading or spelling), as well as other anxiety-related constructs such as self-efficacy and attitudes toward school performance in general and generalized anxiety. Second, the sample size is considerable, but still not enough for an analysis of different genotype groups separately, particularly when considering only the boys or only the girls with the Met/Met genotype. Third, MA is not caused by a single gene, so that many more candidate genes and environmental factors will need to be studied. Fourth, MA probably results from a combination of math-specific factors and general anxiety. The present study has ruled out the likelihood of this gene operating by affecting math ability, but it is not clear as yet whether it could be operating by affecting general anxiety.

Notwithstanding its limitations, the present study adds important information to the knowledge of the neurogenetic underpinnings of MA: (a) a thorough understanding of the origins of MA requires considerations of both environmental and genetic factors; (b) the dopaminergic system, a multifunctional system especially important in human evolution (Previc, 2009; Piffer, 2013), is also relevant for clarifying the neurobiological underpinnings of MA; (c) testing for associations between psychological phenotypes and single-loci genetic markers should consider all possible genetic models (dominance, codominance, and heterosis); and (d) sex differences in MA associated with the *COMT* Val158Met polymorphism are detectable even before puberty. Sex differences in the effect of Val158Met polymorphism in prepubertal children have already been described for cognitive functions (Barnett et al., 2007). Future research should investigate whether the heterosis model of the interaction among sex, *COMT* Val158Met polymorphism and MA is generalizable to other forms of anxiety. An epigenetic research approach is required to address interactions among the *COMT* Val158Met polymorphism with other dopaminergic and non-dopaminergic genes and with sex-hormonal and other metabolic pathways.

## Ethics Statement

This study was carried out in accordance with the recommendations of Resolução no. 196/96 and Resolução 466/12, of the Conselho Nacional de Saúde of the Brazilian Ministério da Saúde. The protocols were approved by the Comitê de Ética em Pesquisa da Universidade Federal de Minas Gerais. All subjects gave written informed consent in accordance with the Declaration of Helsinki.

## Author Contributions

MRSC and VH designed the experiments, supervised data collection and analyses, and wrote the manuscript. GW analyzed results and wrote the manuscript. AM, AJ-C, MA, and MM collected and analyzed data and helped to write the manuscript.

## Conflict of Interest Statement

The authors declare that the research was conducted in the absence of any commercial or financial relationships that could be construed as a potential conflict of interest.
