# Supplementary material for: Heterosis in COMT Val158Met Polymorphism Contributes to Sex-Differences in Children’s Math Anxiety
Source: Front Psychol. 2019 May 15;10:1013. doi: 10.3389/fpsyg.2019.01013 (PMC6530072; doi:10.3389/fpsyg.2019.01013)
Supplement: Supplementary file 1 [file Table_1.pdf]

Table 1: Studies investigating the association between the Val158Met *COMT* polymorphism and anxiety in children and adolescents

Study	Age in years (sample size)	Study design	Genotype groups	Anxiety Measures	Statistical analysis<br>Between groups' difference<br>(effect size, ES)	Sex differences	Genotype by stress interaction
Arbelle et al., 2003	7-8 (n=98)	Cross-sectional	Val/Val, Val/Met, Met/Met	Schedule for Affective Disorders and Schizophrenia for School-Age Children, Martin Temperament Assessment Battery and Achenbach Behavior Checklist	Univariate ANOVA<br>Non significant<br>$\eta^2 = 0.20$	Not investigated	Not investigated
Olsson et al., 2005	14 and 24 (n=962)	Longitudinal<br>2 measures (10 years)	Val/Val, Val/Met, Met/Met	Clinical Interview Schedule-Revised (CIS-R)	Logistic regression and ANOVA<br>Met/Met: higher persistent episodic anxiety<br>ES: Information not available	Met allele effects only in females	Not investigated
Olsson et al., 2007	14 and 24 (n=962)	Longitudinal<br>2 measures (10 years)	Val/Val, Val/Met, Met/Met	Clinical Interview Schedule-Revised (CIS-R)	Logistic regression<br>Double recessive interaction: twofold reduction of persistent generalized anxiety in COMT (Met/Met) and 5HTTLPR (S/S) individuals<br>ES: Information not available	No sex interaction	Genetic protection under high stress conditions positive, not significant
Evans et al., 2009	6-7 (n=8431)	Cross-sectional	Met/Met, Met/ValA, Met/ValB, ValA/ValA, ValA/ValB,	Strengths and Difficulties Questionnaire (SDQ) and Life Event Questionnaire	Logistic regression<br>Non-significant<br>ES: Information not available	No sex interaction	Anxiety and depression in adults; no and COMT genotype by

			ValB/ValB				stress interaction
Gadow et al., 2009	4-14 (n=67)	cross-sectional	Met/-, Val/Val	Child Symptom Inventory-4 (CSI-4)	ANOVAs<br>marginally significant for teacher ratings of social phobia (0.06) (Met/- higher levels)<br>ES: η² = 0.06	Not investigated	Not investigated
Middeldorp et al., 2010	7, 10, 12, 14 and 18; (n=1240)	Longitudinal<br>5 measures (11 years)	Val/Val, Val/Met, Met/Met	Child Behavior Check List (CBCL) at ages 7, 10 and 12; Youth Self Report (YSR) at 14 and 18 years	Factorial association model and path analysis<br>No effect for anxiety or depression<br>ES: Information not available	Not investigated	Authors did not investigate
Shashi et al., 2010	7-16 (n=40)	Cross-sectional	Val/-,<br>Met/-	Child Behavior Checklist (CBCL) and Computerized Diagnostic Interview for Children (C-DISC)	Non-parametric (Mann-Whitney)<br>Val allele associated with higher frequency of anxiety disorders<br>ES: Cohen's d = 1.03	No sex interaction	Not investigated
Sheikh et al., 2013 (Study 1)	~3.5<br>(n=476)	Cross-sectional	Val/Val, Met/-	Preschool Age Psychiatric Assessment (PAPA)	Non-parametric (Mann-Whitney)<br>Val/Val children, higher levels of depressive symptoms<br>ES: Cohen's d = 0.28*	Not investigated	Not investigated

Sheikh et al., 2013 (Study 2)	~3.5(n=409)	cross-sectional	Val/Val, Met/-	Early Childhood Inventory-4 (ECI-4)	Non-parametric (Mann-Whitney)<br>Val/Val children, higher levels of depressive symptoms<br>Cohen's d = 0.29*	Not investigated	Not investigated
Lehto et al., 2013	15, 18 e 25 (n= 593)	longitudinal<br>3 measures<br>(10 years)	Val/Val, Val/Met, Met/Met	Mini-International Neuropsychiatric Interview (MINI 500)	Mixed linear models, ANOVA or ANCOVA<br>Val/Val higher neuroticism scores<br>ES: Information not available	Val/Val effects only in females over age 25	Not investigated
Sheikh et al., 2017	~3 (n=409)	cross-sectional	Val/Val, Met/-	Child Behavior Checklist	Regression-based framework<br>Val/- higher anxiety under stress<br>ES: Cohen's d = 0.15*	Higher depressive and anxious symptoms in girls	Stress, inferred from cortisol levels, moderated the association between COMT Val158Met polymorphism and anxiety

Note: Clinical samples were used in Gadow et al., 2009 (Autism Spectrum Disorder) and in Shashi et al., 2010 (22q11.2 deletion syndrome). Middeldorp et al., 2010 sample includes 288 monozygotyc male twins and 320 monozygotic female twins; 382 male singletons and 392 female singletons.

References

Arbelle S, Benjamin J, Golin M, Kremer I, Belmaker RH, Ebstein RP. Relation of shyness in grade school children to the genotype for the long form of the serotonin transporter promoter region polymorphism. Am J Psychiatry. 2003 Apr;160(4):671-6.

Evans J, Xu K, Heron J, Enoch MA, Araya R, Lewis G, Timpson N, Davies S, Nutt D, Goldman D. Emotional symptoms in children: The effect of maternal depression, life events, and COMT genotype. Am J Med Genet B Neuropsychiatr Genet. 2009 Mar 5;150B(2):209-18. doi: 10.1002/ajmg.b.30789.

Gadow KD, Roohi J, DeVincent CJ, Kirsch S, Hatchwell E. Association of COMT (Val158Met) and BDNF (Val66Met) gene polymorphisms with anxiety, ADHD and tics in children with autism spectrum disorder. J Autism Dev Disord. 2009 Nov;39(11):1542-51. doi: 10.1007/s10803-009-0794-4.

Lehto K, Akkermann K, Parik J, Veidebaum T, Harro J. Effect of COMT Val158Met polymorphism on personality traits and educational attainment in a longitudinal population representative study. Eur Psychiatry. 2013 Oct;28(8):492-8. doi: 10.1016/j.eurpsy.2013.06.008.

Middeldorp CM, Slof-Op 't Landt MC, Medland SE, van Beijsterveldt CE, Bartels M, Willemsen G, Hottenga JJ, de Geus EJ, Suchiman HE, Dolan CV, Neale MC, Slagboom PE, Boomsma DI. Anxiety and depression in children and adults: influence of serotonergic and neurotrophic genes? Genes Brain Behav. 2010 Oct;9(7):808-16. doi: 10.1111/j.1601-183X.2010.00619.x.

Olsson CA, Anney RJ, Lotfi-Miri M, Byrnes GB, Williamson R, Patton GC. Association between the COMT Val158Met polymorphism and propensity to anxiety in an Australian population-based longitudinal study of adolescent health. Psychiatr Genet. 2005 Jun;15(2):109-15.

Olsson CA, Byrnes GB, Anney RJ, Collins V, Hemphill SA, Williamson R, Patton GC. COMT Val(158)Met and 5HTTLPR functional loci interact to predict persistence of anxiety across adolescence: results from the Victorian Adolescent Health Cohort Study. Genes Brain Behav. 2007 Oct;6(7):647-52. Epub 2007 May 14. PubMed PMID: 17504250.

Shashi V, Howard TD, Keshavan MS, Kaczorowski J, Berry MN, Schoch K, Spence EJ, Kwapil TR. COMT and anxiety and cognition in children with chromosome 22q11.2 deletion syndrome. Psychiatry Res. 2010 Jul 30;178(2):433-6. doi:10.1016/j.psychres.2010.04.048.

Sheikh HI, Kryski KR, Kotelnikova Y, Hayden EP, Singh SM. Catechol-O-Methyltransferase gene (val158met) polymorphisms and anxious symptoms in early childhood: The roles of hypothalamus-pituitary-adrenal axis reactivity and life stress. Neurosci Lett. 2017 Oct 17;659:86-91. doi:10.1016/j.neulet.2017.08.060.

Sheikh HI, Kryski KR, Smith HJ, Dougherty LR, Klein DN, Bufferd SJ, Singh SM, Hayden EP. Catechol-O-methyltransferase gene val158met polymorphism and depressive symptoms during early childhood. Am J Med Genet B Neuropsychiatr Genet. 2013 Apr;162B(3):245-52. doi: 10.1002/ajmg.b.32141.
